# Comparison of whole genome amplification techniques for human single cell exome sequencing

**DOI:** 10.1371/journal.pone.0171566

**Published:** 2017-02-16

**Authors:** Erik Borgström, Marta Paterlini, Jeff E. Mold, Jonas Frisen, Joakim Lundeberg

**Affiliations:** 1 Scilifelab, Division of Gene Technology, KTH Royal Institute of Technology, Stockholm, Sweden; 2 CMB, Department of Cell and Molecular Biology, Karolinska Institutet, Stockholm, Sweden; University of North Carolina at Chapel Hill, UNITED STATES

## Abstract

**Background:**

Whole genome amplification (WGA) is currently a prerequisite for single cell whole genome or exome sequencing. Depending on the method used the rate of artifact formation, allelic dropout and sequence coverage over the genome may differ significantly.

**Results:**

The largest difference between the evaluated protocols was observed when analyzing the target coverage and read depth distribution. These differences also had impact on the downstream variant calling. Conclusively, the products from the AMPLI1 and MALBAC kits were shown to be most similar to the bulk samples and are therefore recommended for WGA of single cells.

**Discussion:**

In this study four commercial kits for WGA (AMPLI1, MALBAC, Repli-G and PicoPlex) were used to amplify human single cells. The WGA products were exome sequenced together with non-amplified bulk samples from the same source. The resulting data was evaluated in terms of genomic coverage, allelic dropout and SNP calling.

## Background

Each human tissue is a heterogeneous mix of cells and the genome of each single cell differs from the neighboring cells. Single cell genomics aim to find, analyze and evaluate the importance of these differences. Examples of recent and potential applications are analysis of genetic mosaicism such as cancer heterogeneity [1] and lineage tracing [2]. Furthermore, single cell analysis offers invaluable insights in the field of environmental biology and metagenomics which otherwise would be dependent to cell culture prior to analysis.

Amplification of genetic material is currently a prerequisite prior to whole genome or targeted sequence analysis of single cell genomes. A typical healthy human somatic cell harbors only two copies of the genome, a total of ~6-7pg of double stranded DNA. There are several techniques for amplification and/or sequencing library generation from small amounts of genomic DNA and many of them are available as commercial kits. However, all whole genome amplification (WGA) techniques introduce bias and artifacts compared to unamplified material. Many protocols with different methods for amplification have been published to date [3–8]. Currently, three WGA strategies are widely used for: single cell comparative genomic hybridization (SCOMP), multiple displacement amplification (MDA) and a combination of displacement pre-amplification and PCR amplification (marked as Picoplex and MALBAC). The protocols may thus display different rates of artifact formation, allelic dropout and loss of sequence coverage over the genome. Choosing among different amplification techniques to minimize dropout and preferential amplification, increase genome coverage or maximize base replication accuracy can therefore be of great importance.

WGA techniques have previously been compared for genotyping, whole genome and exome sequencing of samples with low amounts of input material [9–12]. Comparisons for single cell sequencing targeting bacterial [13] and human [14] genomes have also been performed recently.

To facilitate the choice of amplification technique for human single cell sequencing we compared four of the most frequently used and commercially available WGA kits; AMPLI1, MALBAC, Repli-G and PicoPlex (S1 Fig). Differently from previous comparisons [13,14], we analyze one kit more, that is, AMPLI1. The AMPLI1 kit works by fragmentation of the genome by a set of restriction enzymes and ligation of adapters to the resulting sticky ends followed by exponential temperature cycled amplification primed by the attached adapter sequences. The method is based on the SCOMP method published in the end of the 1990s [4,15]. The multiple displacement amplification (MDA) method described in 2002 utilize phi29 DNA polymerase to do isothermal amplification^5^. Due to the high fidelity of phi29 MDA methods usually display low error rates but the non-linear amplification makes it more prone to preferentially amplify some regions [16]. REPLI-g is one of several commercially available MDA based WGA kits. MALBAC and PicoPlex both initially perform a “quasi linear” pre-amplification using degenerate primers with handles attached to the 3’-end [3,17]. This accumulates a looped or hairpin library and is followed by exponential amplification with primers hybridizing to handle sequences. Due to the linear part of the amplification these kits have been shown to produce more even coverage^3^. However, for the MALBAC kit this has been at the expense of fidelity as a non-proofreading polymerase is used in the preamplification [1].

In the comparison presented here whole genome amplification of two human single cells for each of the kits described above was performed. This was followed by library preparation, exome sequencing and evaluation of the resulting data in terms of genomic coverage, allelic dropout and SNP calling.

## Results

**Fig 1:**
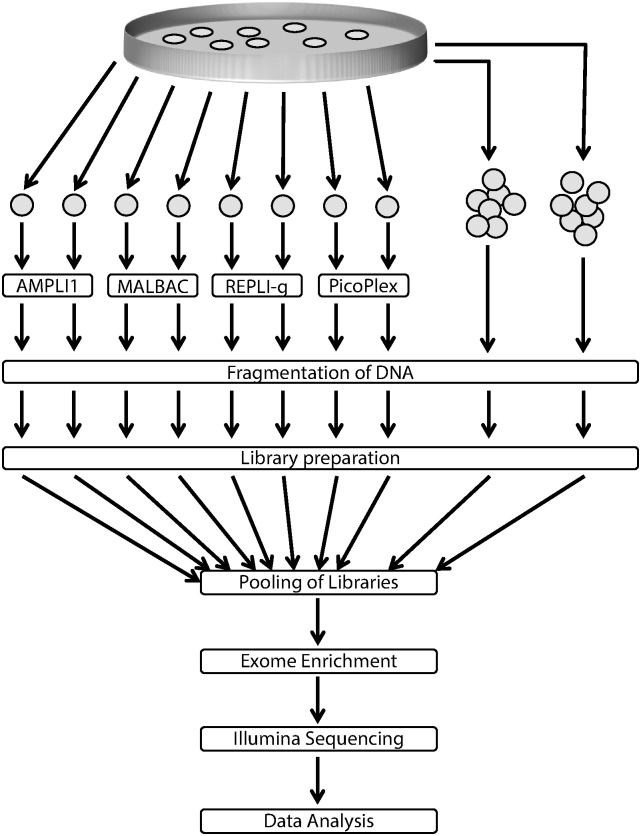
Overview of the experimental workflow: Single HepG2 cells were isolated and whole genome amplification was performed using four commercially available kits. The WGA products as well as two unamplified bulk samples were fragmented by sonication and sequencing libraries were prepared. Exome enrichment was performed after pooling of the libraries. Finally the enriched pools were sequenced and the resulting data analyzed.

To develop our comparison on single-cell sequencing methodologies, single cells of HepG2 cells were isolated. Four commonly used whole genome amplification kits; AMPLI1, MALBAC, Rubicon PicoPlex and REPLI-g were compared. Each method was used to amplify a pair of single cells and sequencing libraries were created from the eight resulting WGA products as well as genomic DNA extracted from two bulk samples (also from the same cell culture). The libraries were enriched for molecules carrying exome sequence and sequenced using the Illumina sequencing technology (see Fig 1 for an overview of the workflow). The genomic integrity of fragmented WGA products were also analyzed using qPCR targeting 16 amplicons as described earlier [3] S2 Fig).

Identical amounts of input material were used at each part of the protocol (library preparation, exome enrichment and sequencing). However the output of data acquired from the different WGA products differed significantly (S1 Table).

Accordingly, subsets of one to ten million reads were randomly chosen from each of the libraries. It was observed that the exome coverage reached a plateau after ten million reads (S3 Fig). To enable direct comparison of read mapping and target coverage statistics, all the following results are based on the ten million read pair subset.

**Fig 2:**
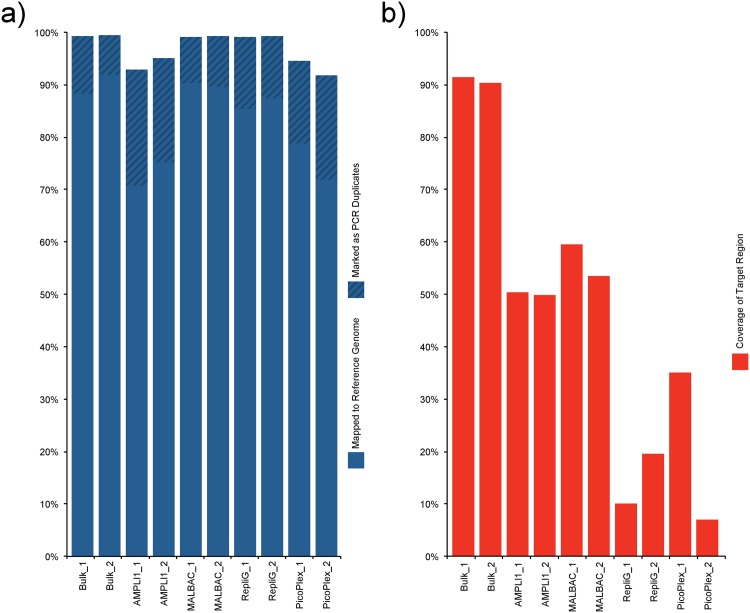
For each of the different samples in the 10M read pair subset, mapping rates are homogenous among samples over 90% (a), and exome coverage (b) was considerably lower for the amplified single cells (ranging from 7 to 68%) compared to the bulk samples (~90% coverage).

The rates of reads mapping to the reference genome were similar for all samples, above 90%. However, the bulk samples had, as expected, higher rates than any of the single cell samples. The MALBAC and REPLI-g samples showed slightly higher rates than AMPLI1 and PicoPlex (Fig 2a). AMPLI1 and PicoPlex amplified samples exhibited slightly higher rates of PCR duplicates compared to other samples (Fig 2a, S1 Table). GC content distributions of the read population resembled the Bulk Samples for all WGA products except the MALBAC products that showed a slightly higher average GC (S4 Fig).

The target coverage was considerably lower for the amplified single cells (ranging from 7 to 68%) compared to the bulk samples (~90% coverage). Generally reflecting the results from the genomic integrity qPCR (S2 Fig). Furthermore, the proportion of reads mapping outside the targeted region is much larger for the worst of the WGA products (54%, AMPLI1_1) compared to the best bulk sample (14%, BULK_2) (S1 Table). The amount of WGA adapters sequenced (and trimmed) also influenced this result as the percentage of bases trimmed differed between WGA procedures (S2 Table). In terms of target coverage AMPLI1 and MALBAC perform far better than REPLI-g and PicoPlex which only cover minor fractions of the target (Fig 2b, S2 Fig). The two replicates of the PicoPlex amplification also show a large variation in target coverage. The genomic integrity analysis by qPCR was therefore performed on seven additional cells amplified with the PicoPlex WGA kit to assess the rate of success for this method (S5 Fig). Out of the seven tested cells no WGA product amplified all target amplicons in the same Ct range as the positive controls and only one were positive for more than half of the amplicons. Confirming variable success rate of the PicoPlex reaction.

**Fig 3:**
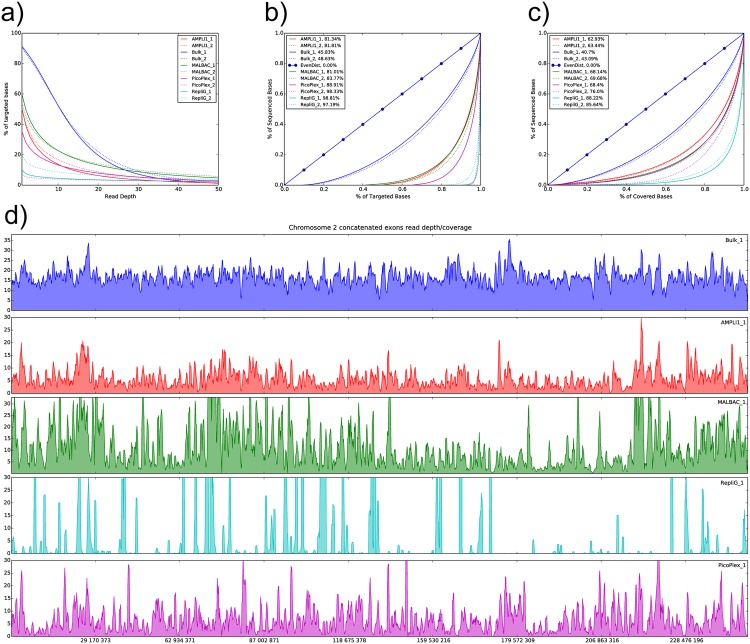
Percent target coverage for each of the samples in the 10M read pair subset at various read depths (a). Lorenz curves and Gini indexes describing how sequenced bases are distributed over targeted (b) and covered (c) regions and showing an uneven distribution of read over the targeted regions. Read depth of 1kb sliding windows visualized over chromosome 2 for one sample of each of the different WGA kits as well as the Bulk_1 sample (d).

The low targeted region coverage observed for PicoPlex and REPLI-g, in conjunction with mapping rates that are comparable to the other single cell WGAs, indicate that there is an uneven distribution of reads over the targeted regions. This was confirmed by evaluating the coverage at varying read depth and by generating Lorenz curves showing the coverage distribution and calculating the Gini index for the bulk samples and each of the single cell WGAs (Fig 3a, 3b and 3c). Single base read depth histograms were generated and compared to the Poisson distribution for each sample (S6 Fig). Generally showing larger than expected proportions of bases with read depth zero as well as exaggerated read depth also indicating uneven amplification of the targeted regions. The result was once more confirmed by visualization of read depth plotted over the targeted regions of chromosome 2 in sliding 1 kb windows (Fig 3d).

**Fig 4:**
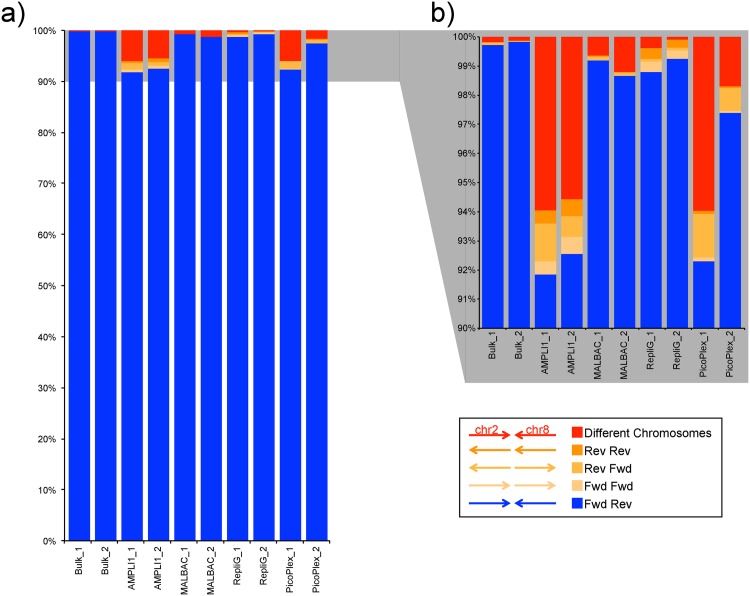
Read pair mapping orientations (fwd, fwd; rev, fwd; or rev, rev) as percentage of total number of read pairs for each sample in the 10M read pair subset. The right panel shows an enlarged version of the region from 90% to 100%. All samples showed >90% of read pairs mapping in the expected directions.

Next, we investigated the concordance of mapped read pairs. The two reads in a pair from a standard Illumina Paired End (PE) library are expected to map in the forward followed by reverse (fwd, rev) manner. Any other way of mapping (ie fwd, fwd; rev, fwd; or rev, rev) or when the two reads from one pair map to different chromosomes indicates either a variation within the genome or formation of artifacts during WGA or library preparation. All samples showed >90% of read pairs mapping in the expected directions (Fig 4). Still single cell WGAs resulted in higher rates of unexpected mapping orientations compared to the bulk samples. AMPLI1 showed the overall poorest performance in terms of such artifact formation (up to 8% of the reads, S3 Table).

**Fig 5:**
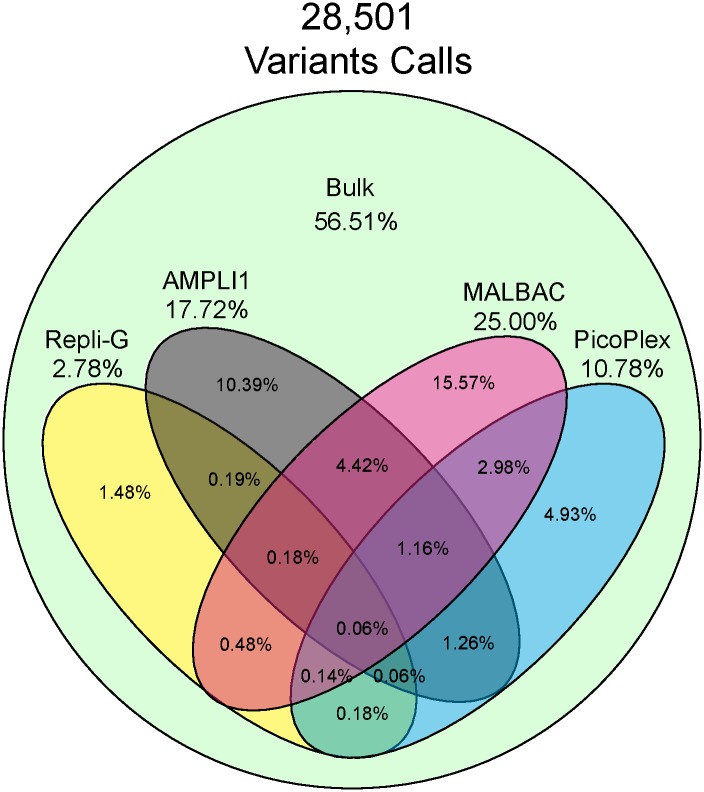
Venn diagram showing the overlap of variant calls in the different samples compared to the 28k variants called in the Bulk_1 sample, compared to the human reference genome (GRCh37): 25% of the bulk sample variants were called correctly in the MALBAC-data. For AMPLI1, Picoplex and Repli-G the value was 18%, 11% and 3% respectively.

Variants were called compared to the human reference genome (GRCh37), and the concordance of called variants between the different WGA samples and one of the bulk samples were evaluated (Fig 5, S4 Table). By using the Bulk variant calls as a reference set we remove the effects of single cell heterogeneity might have on the results. Approximately 25% of the bulk sample variants were called correctly in the MALBAC-data. For AMPLI1, Picoplex and Repli-G the result was 18%, 11% and 3% respectively. Due to varying target coverage the total number of called variants for each sample differed significantly. When evaluating the rate of called variants that were called correctly for each sample, the numbers were 96%, 81%, 62% and 52% for AMPLI1, MALBAC, Picoplex and Repli-G respectively.

**Fig 6:**
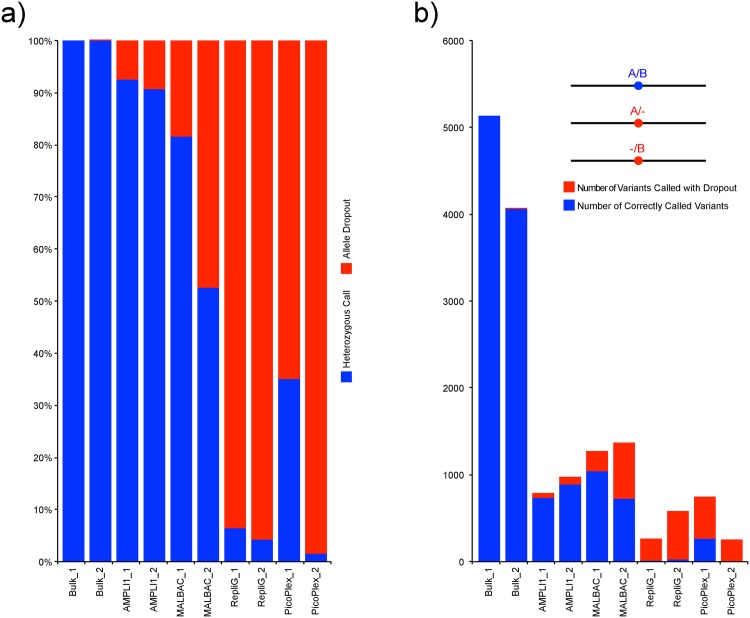
Allelic dropout of SNVs called as heterozygous in the Bulk_1 sample. Both normalized to the total number of variants that overlap with the Bulk_1 sample (a) and variant counts (b).

An estimate of allele dropout (ADO) can be obtained by evaluating the rate of variants called as heterozygous in the bulk sample though called as homozygous in WGA samples. As expected the bulk sample replicates showed close to full overlap of heterozygous SNP calls (99.9%). For the single cell WGAs AMPLI1 most often displayed representation of both alleles within the called set of variants (up to 92.5%, Fig 6a). Repli-G on the other hand mostly amplified only one of the two alleles (S5 Table). MALBAC and PicoPlex both showed large variability between the duplicates. Furthermore the total number of positions that overlapped with the bulk sample varied considerably among the single cells (Fig 6b). The positions of the analyzed variants were plotted along with the read depth visualizations over the chromosomes (S7 Fig compared to Fig 3d) to assess if dropout occurred in specific areas. The result indicate that allele drop out is spread over the full length of the chromosome and not restricted to any specific region.

## Discussion

In our analysis we took into consideration more methodologies applied to human cells than previous comparisons [13,14]. We initially investigated basic library characteristics such as output read counts, mapping rates and read mapping distribution and coverage over the target regions. Total read counts for each library differed significantly, which may indicate how accurate the library quantification was as well as how efficient the library was clustered on the flow cell surface in comparison with the other libraries present.

Mapping rates reflect the proportion of library molecules originating from human genomic DNA fragments. All libraries showed comparable and high mapping rates, in agreement with de Bourcy et al. (2014). We therefore conclude that all the methods are relatively robust in terms of formation of artifacts and did not suffer from excessive contamination of non-human sequences. The PCR duplication rates were also comparable for most of the samples. Both the AMPLI1 WGA products and one of the PicoPlex libraries show slightly higher duplication rates. Note that due to the initial fragmentation step performed as part of the Illumina library preparation the duplication rate does not reflect the complexity of the original WGA product. These duplication rates are therefore a measurement of how many molecules of the fragmented WGA product that successfully passed through the library preparation and enrichment by hybridization procedure.

There are large differences in how much of the targeted region is covered by reads for each WGA sample. The bulk samples both reach approximately 90% target coverage, while MALBAC (the best of the single cell WGAs) reaches less than 60% coverage. The target coverage also decreases rapidly if higher read depth is required, indicating large regions of shallow read depth. For the AMPLI1 and PicoPlex kits some of the difference in target coverage compared to bulk and other WGAs is explained by lower proportion of the reads mapping to the targeted regions. This could be explained by the shorter insert size for both those libraries (S8 Fig) and non random fragmentation procedure in the AMPLI1 case, leading to less efficient hybridization of capture probes to the library molecules. Also note that up to 14% of the sequenced bases for each single cell WGA is spent on reading the ligated adapters or handle sequences attached to the primer used for amplification. The WGA samples are therefore not expected to reach the same genome coverage as the bulk samples. Still, for the Repli-G amplification that displays the least target coverage no adapter sequences are expected within the read pairs. Evaluation of the distribution of reads over targeted regions (Fig 3b) and covered regions (Fig 3c) confirm that all four WGA methods have significant amounts of uncovered regions and show more uneven read depth distributions over the covered regions than the unamplified samples. Repli-G shows the most uneven distribution and most uncovered regions followed by PicoPlex. MALBAC shows the most even read distribution, as described in de Bourcy et al. (2014) [13] and Cheng et al. (2014) [14] as well, and fewest uncovered regions although these are clearly separated from the bulk samples. The AMPLI1 kit covers a slightly less of the target compared to the MALBAC WGA though has a slightly more even distribution of read depth over the covered bases. AMPLI1 could potentially perform better for whole genome sequencing (WGS) than for exome sequencing as the target enrichment by hybridization was less efficient. Unexpectedly one of the PicoPlex replicates exhibits statistics that are comparable to the Repli-G samples, while the other replicate is closer to the method-wise more similar MALBAC and AMPLI1 kits (S1 Fig). This may be explained by a failed WGA reaction that for some reason only amplified a subset of the genome as indicated by the genomic integrity qPCR. This was observed once more, when only one of the additional PicoPlex reactions was successful for a majority of the targets indicating a variable success rate for PicoPlex amplification. MDA based amplification, such as the Repli-G WGA, has earlier been shown to be sensitive to reaction gain^13^. High gain potentially leads to a strong bias towards certain genomic regions and a reaction gain lower than the manufacturer's instruction (or another MDA based kit) might thus perform significantly better. For application where high and even target coverage is essential AMPLI1 or MALBAC should be used in favor of the REPLi-G or PicoPlex kits.

The proportion of read pairs mapping in unexpected directions or on different chromosomes were generally less than 10% of the data. For the two bulk replicates more than 99.5% of the reads mapped in the expected direction and less than 0.2% mapped on different chromosomes. Comparable rates were observed for all WGA libraries except the AMPLI1 samples and one of the two PicoPLex replicates (surprisingly not the potentially failed WGA replicate with the lower target coverage). These samples showed comparable proportions for most types of unexpected mapping directions. However, all three of the samples displayed more than 5% read pairs where the two reads mapped to different chromosomes. In the case of AMPLI1 WGA this might be caused by the restriction enzyme based fragmentation followed by ligation of adapters to the fragments with a risk of ligating two genomic fragments to each other instead of adapters. For the PicoPlex WGA on the other hand this was more unexpected. This indicates that extra consideration might be necessary when calling of structural variants (based on mapping orientations) from single cells amplified with the AMPLI1 kit.

Out of the 28,501 variants that were called in the bulk sample and passed filtering, 57% were found only in the bulk sample and did not pass filters for any other sample. MALBAC was able to call the largest proportion of the variants compared to the other WGA methods. It is likely that this is to a large extent due to the larger proportion of the reads being successfully mapped to the target regions, giving a greater read depth and resulting in variants passing the filter more often than for the other methods. AMPLI1 gives the highest rate of correct calls (96%) when normalizing for the total number of called variants followed by MALBAC (81%), Picoplex (62%) and lastly Repli G (52%). Note that these results cannot be interpreted as only polymerase errors as some of the erroneous genotype calls are due to ADO. As expected almost full overlap of heterozygous calls were observed for the two bulk samples. All of the WGAs however showed homozygous calls at positions where heterozygous calls were observed in the bulk samples. This indicates allelic dropout in these sample either due to preferential amplification or loss of one molecule in the WGA reaction. Differences in mapping efficiencies should not be profound as each variant corresponds to a heterozygous call in one of the bulk samples. The extremes AMPLI1 and Repli-G called >90% resp. <10% of the expected heterozygous variants correctly. The total numbers of variants expected to be heterozygous (based on bulk sample data) differed significantly for the different WGAs (Fig 6b). A high ADO rate should lead to low target coverage as evident in the case for the Repli-G WGA. Of the WGAs tested MALBAC produced most correct genotype calls overlapping with variations present in the bulk sample, much due to the high target coverage. AMPLI1 display the highest rate of correct variant calls to some extent due to the lowest observed ADO rate. Repli-G and PicoPlex both display higher rates of ADO and therefore also fewer correct genotype calls.

In summary four commercially WGA kits have been tested according to manufacturer's protocols and the products were applied to exome enrichment and sequencing. Considering the evaluated statistics AMPLI1 or MALBAC would be the recommended WGA for”out of the box” usage as they display the highest fidelity for both exome coverage and variant calling compared to the bulk samples.

## Methods

### DNA samples

We compared the state of art of Whole Genome Amplification methods available on the market. Protocols compared in this study were independently tested on human genomic DNA samples of human hepatocellular liver carcinoma cell line (HepG2). HepG2 cells were cultured on tissue culture plates in Dulbecco’s modified Eagle’s medium (DMEM) containing 10% fetal bovine serum (FBS). After 4 days, the adherent cells reached 70–80% confluence. Cells were dissociated by trypsine-EDTA, counted and diluted to a concentration of 1 cell/μl by serial dilution. From a final 200 μl suspension at this concentration, one microliter was seeded in each well in a flat 96-well culture microplate. Wells were checked one by one under the light microscope and drops with a single cell only were marked.

### Whole genome amplification

The Whole Genome Amplification (WGA) was carried out only on the labeled single cells wells. WGA was performed using the commercially available: PicoPlex (Rubicon), REPLI-g Mini Kit (Qiagen), Multiple Annealing and Looping Based Amplification Cycles (MALBAC, Yikongenomics, China), and AMPLI1 (Silicon Biosystems). All WGA reactions were carried out according to the manufacturer's instructions. The resulting amplified DNA from the REPLI-G, MALBAC and AMPLI1 products was purified using the Qiaquick PCR Purification Kit (Qiagen, Canada), and DNA from PicoPlex was purified using the DNA Clean & Concentrator-5 kit (Zymo Research). Cleaned-up products were quantified with Quibit fluorometer using a dsDNA BR kit (Invitrogen, Q32853) and their resulting size distribution was checked using the Agilent 2100 Bioanalyzer DNA 1000 or High sensitivity assays. Duplicate single cells were amplified for all techniques.

### Libraries preparation and enrichment and sequencing

Libraries were prepared from 250 ng Covaris-sheared DNA, using the TruSeq DNA LT Sample Prep Kit and a previously described automated protocol[18] modified to be compatible with Illumina TruSeq library preparation protocols and reagents. Afterwards, libraries were pooled and Illumina TruSeq Exome Enrichment were performed for according to manufacturer's protocols. Sequencing of the enriched libraries were performed on Illumina HiSeq (2x100bp, Rapid mode) and Illumina MiSeq (2x150bp).

### Data analysis

The read pairs from the WGAs and Bulk samples were trimmed either by a custom script or Cutadapt[19] to remove any WGA adapter sequences. Cutadapt were also used to trim Illumina adapter sequences and TrimBWAstyle.pl[20] to remove low quality base calls. The trimmed read pairs were then quality assessed using FastQC[21] and aligned to the human genome (GRcH37) using bowtie2[22]. Mapped read pairs were and filtered for PCR duplicates using Picard Tools[23] and any paired not marked as “Proper Pair” by bowtie or having a mapping quality less than 20 were removed using Samtools[24]. Bedtools[25] was used to generate the coverage statistics. Variants were called and filtered using the Genome Analysis ToolKit[26] (GATK) according to the “Best Practices” guidelines[27,28]. As described in the best practices guidelines, known sites from the GATKBundle were used for the indel realignment, base recalibration, haplotype calling and variant recalibration steps. All variant genotype comparisons were made using custom scripts and only included variant genotype calls where sample read depth (DP) was equal to or greater than 10 and the genotype quality (GQ) was equal to or greater than 30. Coverage and variant calling analysis were limited to the targeted regions. All scripts used for the analysis can be found at https://github.com/elhb/singleFatCellExomeAnalysis/tree/fnuttglugg.

## Supporting information

S1 Fig Schematic overview of the four kits used for whole genome amplification. (PDF)

S2 Fig Genomic integrity qPCR of seven PicoPlex WGA products. For each sample the bars show the sample Ct as a percentage value for the 16 amplicons (0% corresponds to the Ct for the negative control and 100% corresponds to the Ct for the positive control). Green bars have values above 66.7%, yellow bars are between 33.3% and 66.7% while the red bars have values below 33.3%.
(PDF)

S3 Fig The percentage of exome coverage observed for subsets of one to ten million read pairs for each of the libraries. (PDF)

S4 Fig GC distributions for the read one and read two populations for each WGA product and the Bulk samples. Showing a slightly higher mean GC % for the MALBAC products, all other samples closely match the Bulk samples.
(PDF)

S5 Fig Genomic integrity qPCR of fragmented WGA products. For each sample the bars show the sample Ct as a percentage value for the 16 amplicons (0% corresponds to the Ct for the negative control and 100% corresponds to the Ct for the positive control). Green bars have values above 66.7%, yellow bars are between 33.3% and 66.7% while the red bars have values below 33.3%.
(PDF)

S6 Fig Single base read depth histogram compared to theoretical Poisson distribution for each sample. (PDF)

S7 Fig Location of the heterozygous and ADO calls in WGAs and the Bulk_1 sample on chromosome 2. (PDF)

S8 Fig Insert sizes of mapped and filtered read pairs in the 10 million subset. (PDF)

S1 Table Amount of data obtained, mapping rates, percentage of reads that map to target regions, rate of PCR duplicates and percentage of the exome covered for each sample in the full data set and the 10M read pair subset. Despite identical amounts of initial material, the output data differed significantly. The target coverage was much lower for the amplified single cells (range between 7 and 68%) compared to the 90% coverage of the bulk.
(PDF)

S2 Table Details results of adapter and quality trimming of reads for each sample in the 10M read pair subset. (PDF)

S3 Table Mapping orientations of read pairs for each sample in the 10M read pair subset. (PDF)

S4 Table Variant and genotype calls compared to the Bulk_1 sample for the first duplicate of all samples in the 10M read pair subset. (PDF)

S5 Table Allele dropout estimation based on variant calls compared to the Bulk_1 sample for each sample in the 10M read pair subset. (PDF)

## Abbreviations

ADO: Allelic Drop Out
DMEM: Dulbecco’s modified Eagle’s medium
DNA: Deoxyribonucleic Acid
EDTA: Ethylenediaminetetraacetic Acid
FBS: Fetal Bovine Serum
GATK: The Genome Analysis Toolkit
GRcH37: Genome Reference Consortium Human release 37
HepG2: Human Hepatocellular Liver Carcinoma Cell Line
MALBAC: Multiple Annealing and Looping Based Amplification Cycles
MDA: Multiple Displacement Amplification
PCR: Polymerase Chain Reaction
SCOMP: Single Cell Comparative Genomic Hybridization PCR
WGA: Whole Genome Amplification
WGS: Whole Genome Sequencing

## References

[1] Navin NE. The first five years of single-cell cancer genomics and beyond. Genome Res. 2015; 1499–1507. 10.1101/gr.191098.115 26430160 PMC4579335

[2] Evrony GD, Lee E, Mehta BK, Benjamini Y, Johnson RM, Cai X, et al Cell Lineage Analysis in Human Brain Using Endogenous Retroelements. Neuron. 2015;85: 49–59. 10.1016/j.neuron.2014.12.028 25569347 PMC4299461

[3] Zong C, Lu S, Chapman AR, Xie XS. Genome-wide detection of single-nucleotide and copy-number variations of a single human cell. Science. 2012;338: 1622–6. 10.1126/science.1229164 23258894 PMC3600412

[4] Klein CA, Schmidt-Kittler O, Schardt JA, Pantel K, Speicher MR, Riethmüller G. Comparative genomic hybridization, loss of heterozygosity, and DNA sequence analysis of single cells. Proc Natl Acad Sci U S A. 1999;96: 4494–9. 10200290 10.1073/pnas.96.8.4494 PMC16360

[5] Dean FB, Hosono S, Fang L, Wu X, Faruqi aF, Bray-Ward P, et al Comprehensive human genome amplification using multiple displacement amplification. Proc Natl Acad Sci U S A. 2002;99: 5261–5266. 10.1073/pnas.082089499 11959976 PMC122757

[6] Langmore JP. Rubicon Genomics, Inc. 2002;3: 557–560. 10.1517/14622416.3.4.557 12164778

[7] Telenius H, Carter NP, Bebb CE, Nordenskjöld M, Ponder B a, Tunnacliffe a. Degenerate oligonucleotide-primed PCR: general amplification of target DNA by a single degenerate primer. Genomics. 1992;13: 718–725. 1639399 10.1016/0888-7543(92)90147-k

[8] Zhang L, Cui X, Schmitt K, Hubert R, Navidi W, Arnheim N. Whole genome amplification from a single cell: implications for genetic analysis. Proc Natl Acad Sci U S A. 1992;89: 5847–51. 1631067 10.1073/pnas.89.13.5847 PMC49394

[9] Pinard R, de Winter A, Sarkis GJ, Gerstein MB, Tartaro KR, Plant RN, et al Assessment of whole genome amplification-induced bias through high-throughput, massively parallel whole genome sequencing. BMC Genomics. 2006;7: 216 10.1186/1471-2164-7-216 16928277 PMC1560136

[10] Uda A, Tanabayashi K, Fujita O, Hotta A, Yamamoto Y, Yamada A. Comparison of whole genome amplification methods for detecting pathogenic bacterial genomic DNA using microarray. Jpn J Infect Dis. 2007;60: 355–361. 18032834

[11] Bergen AW, Haque K a., Qi Y, Beerman MB, Garcia-Closas M, Rothman N, et al Comparison of yield and genotyping performance of multiple displacement amplification and OmniPlex^™^ whole genome amplified DNA generated from multiple DNA sources. Hum Mutat. 2005;26: 262–270. 10.1002/humu.20213 16086324

[12] Hasmats J, Gréen H, Orear C, Validire P, Huss M, Käller M, et al Assessment of whole genome amplification for sequence capture and massively parallel sequencing. PLoS One. 2014;9: 1–10. 10.1371/journal.pone.0084785 PMC3883664 24409309

[13] de Bourcy CF a, De Vlaminck I, Kanbar JN, Wang J, Gawad C, Quake SR. A quantitative comparison of single-cell whole genome amplification methods. PLoS One. 2014;9: e105585 10.1371/journal.pone.0105585 25136831 PMC4138190

[14] Chen M, Song P, Zou D, Hu X, Zhao S, Gao S, et al Comparison of Multiple Displacement Amplification (MDA) and Multiple Annealing and Looping-Based Amplification Cycles (MALBAC) in Single-Cell Sequencing. Zheng D, editor. PLoS One. Public Library of Science; 2014;9: e114520. 10.1371/journal.pone.0114520 PMC4259343 25485707

[15] Klein CA, Blankenstein TJ, Schmidt-Kittler O, Petronio M, Polzer B, Stoecklein NH, et al Genetic heterogeneity of single disseminated tumour cells in minimal residual cancer. Lancet. 2002;360: 683–689. 10.1016/S0140-6736(02)09838-0 12241875

[16] Lage JM, Leamon JH, Pejovic T, Hamann S, Lacey M, Dillon D, et al Whole genome analysis of genetic alterations in small DNA samples using hyperbranched strand displacement amplification and array-CGH. Genome Res. 2003;13: 294–307. 10.1101/gr.377203 12566408 PMC420367

[17] PicoPLEX WGA kit technology. [Internet]. [cited 1 Jan 2015]. http://rubicongenomics.com/products/picoplex/

[18] Borgström E, Lundin S, Lundeberg J. Large scale library generation for high throughput sequencing. PLoS One. 2011;6: e19119 10.1371/journal.pone.0019119 21589638 PMC3083417

[19] Martin M. Cutadapt removes adapter sequences from high-throughput sequencing reads. EMBnet.journal. 2011;17: 10.

[20] http://wiki.bioinformatics.ucdavis.edu/index.php/TrimBWAstyle.pl [Internet]. [cited 1 Jan 2015]. http://wiki.bioinformatics.ucdavis.edu/index.php/TrimBWAstyle.pl

[21] Andrews S. A quality control tool for high throughput sequence data. 2010; http://www.bioinformatics.babraham.ac.uk/projects/fastqc/

[22] Langmead B, Salzberg SL. Fast gapped-read alignment with Bowtie 2. Nat Methods. Nature Research; 2012;9: 357–359. 10.1038/nmeth.1923 PMC3322381 22388286

[23] http://picard.sourceforge.net [Internet]. [cited 1 Jan 2015]. http://picard.sourceforge.net

[24] Li H, Handsaker B, Wysoker A, Fennell T, Ruan J, Homer N, et al The Sequence Alignment/Map format and SAMtools. Bioinformatics. 2009;25: 2078–2079. 10.1093/bioinformatics/btp352 19505943 PMC2723002

[25] Quinlan AR, Hall IM. BEDTools: a flexible suite of utilities for comparing genomic features. Bioinformatics. 2010;26: 841–842. 10.1093/bioinformatics/btq033 20110278 PMC2832824

[26] McKenna A, Hanna M, Banks E, Sivachenko A, Cibulskis K, Kernytsky A, et al The Genome Analysis Toolkit: a MapReduce framework for analyzing next-generation DNA sequencing data. Genome Res. Cold Spring Harbor Laboratory Press; 2010;20: 1297–303. 10.1101/gr.107524.110 PMC2928508 20644199

[27] DePristo MA, Banks E, Poplin R, Garimella KV, Maguire JR, Hartl C, et al A framework for variation discovery and genotyping using next-generation DNA sequencing data. Nat Genet. Nature Research; 2011;43: 491–498. 10.1038/ng.806 PMC3083463 21478889

[28] Van der Auwera GA, Carneiro MO, Hartl C, Poplin R, del Angel G, Levy-Moonshine A, et al From FastQ Data to High-Confidence Variant Calls: The Genome Analysis Toolkit Best Practices Pipeline [Internet]. Current Protocols in Bioinformatics. 2013. 10.1002/0471250953.bi1110s43 PMC4243306 25431634

