# Supplementary material for: Comparison of whole genome amplification techniques for human single cell exome sequencing
Source: PLoS One. 2017 Feb 16;12(2):e0171566. doi: 10.1371/journal.pone.0171566 (PMC5313163; doi:10.1371/journal.pone.0171566)
Supplement: S1 Fig — (PDF) [file pone.0171566.s001.pdf]

	Pre Amp	PCR	Time	ISO thermal	Restriction Enzyme
AMPLI1		√	Two days		√
MALBAC	√	√	Half a day		
RepliG			Overnigth	√	
PicoPlex	√	√	Half a day		

Supplementary Figure 1.
Schematic overview of the four kits used for whole genome amplification.
