# Supplementary material for: Comparison of whole genome amplification techniques for human single cell exome sequencing
Source: PLoS One. 2017 Feb 16;12(2):e0171566. doi: 10.1371/journal.pone.0171566 (PMC5313163; doi:10.1371/journal.pone.0171566)
Supplement: S2 Fig — For each sample the bars show the sample Ct as a percentage value for the 16 amplicons (0% corresponds to the Ct for the negative control and 100% corresponds to the Ct for the positive control). Green bars have values above 66.7%, yellow bars are between 33.3% and 66.7% while the red bars have values below 33.3%. (PDF) [file pone.0171566.s002.pdf]

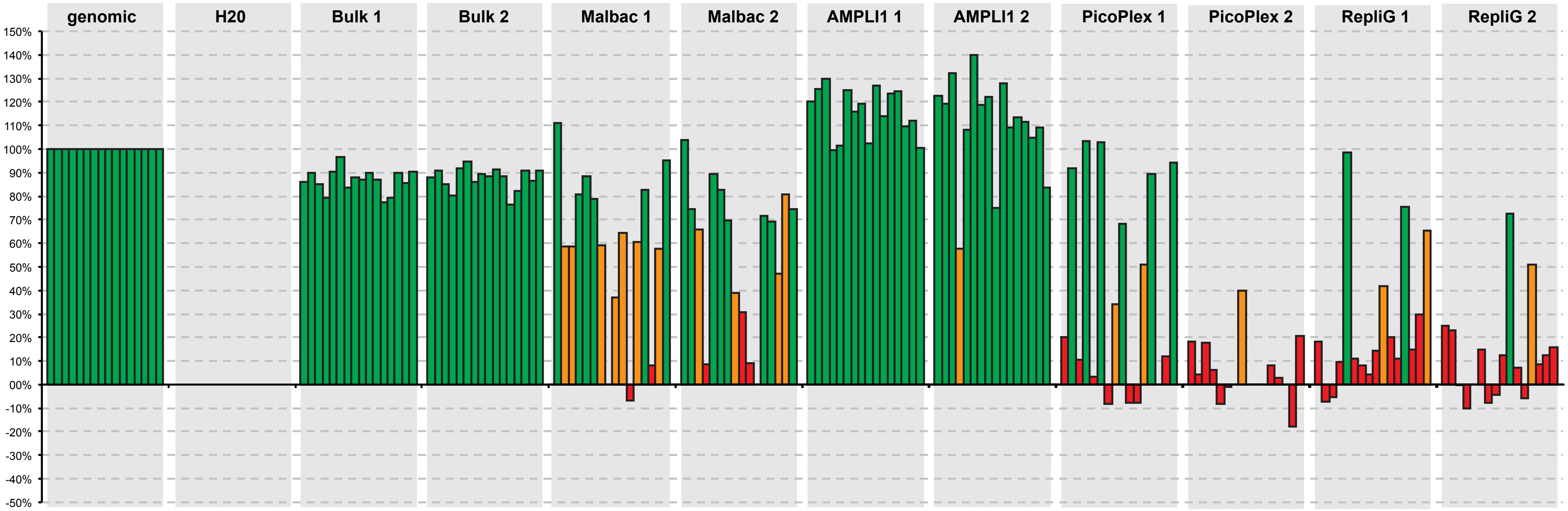

Supplementary Figure 2.
Genomic integrity qPCR of seven PicoPlex WGA products. For each sample the bars show the sample Ct as a percentage value for the 16 amplicons (0% corresponds to the Ct for the negative control and 100% corresponds to the Ct for the positive control). Green bars have values above 66.7%, yellow bars are between 33.3% and 66.7% while the red bars have values below 33.3%.
