# Supplementary material for: Comparison of whole genome amplification techniques for human single cell exome sequencing
Source: PLoS One. 2017 Feb 16;12(2):e0171566. doi: 10.1371/journal.pone.0171566 (PMC5313163; doi:10.1371/journal.pone.0171566)
Supplement: S4 Fig — Showing a slightly higher mean GC % for the MALBAC products, all other samples closely match the Bulk samples. (PDF) [file pone.0171566.s004.pdf]

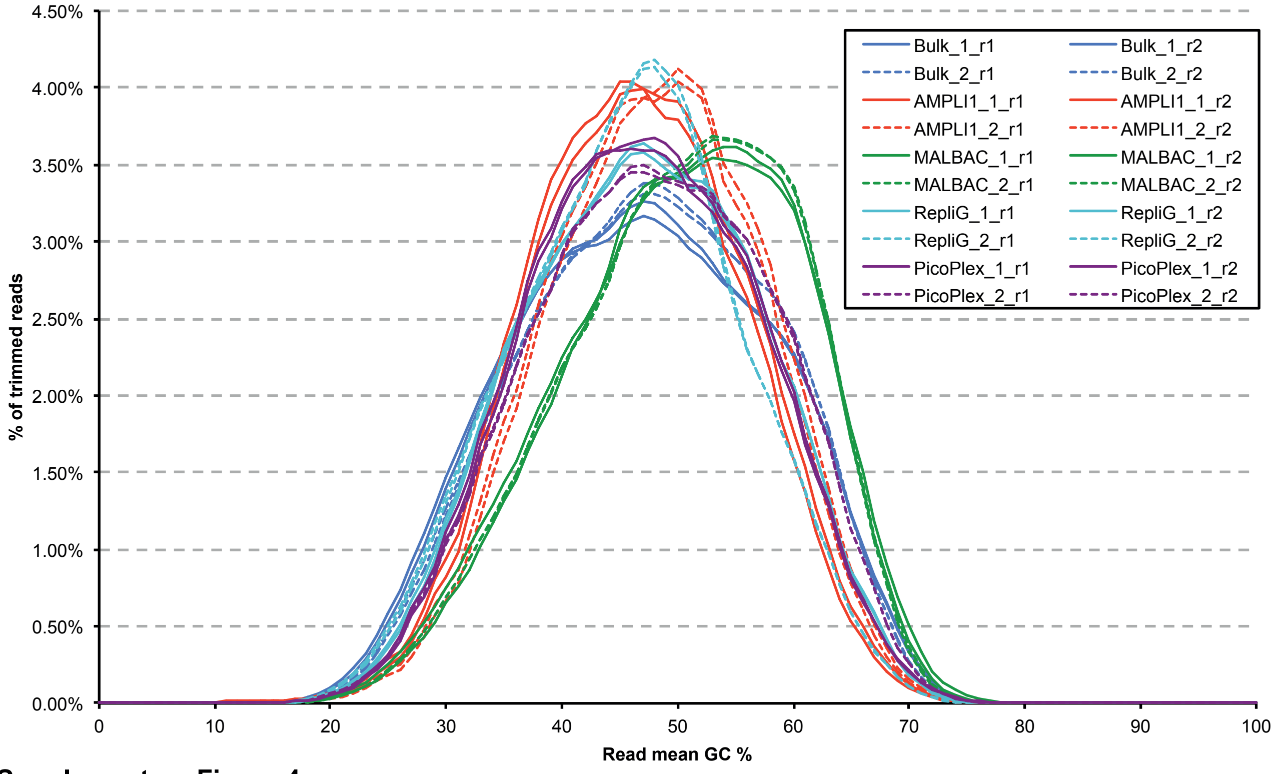

Supplementary Figure 4.

GC distributions for the read one and read two populations for each WGA product and the Bulk samples. Showing a slightly higher mean GC % for the MALBAC products, all other samples closely match the Bulk samples.
