# Supplementary material for: Comparison of whole genome amplification techniques for human single cell exome sequencing
Source: PLoS One. 2017 Feb 16;12(2):e0171566. doi: 10.1371/journal.pone.0171566 (PMC5313163; doi:10.1371/journal.pone.0171566)
Supplement: S7 Fig — (PDF) [file pone.0171566.s007.pdf]

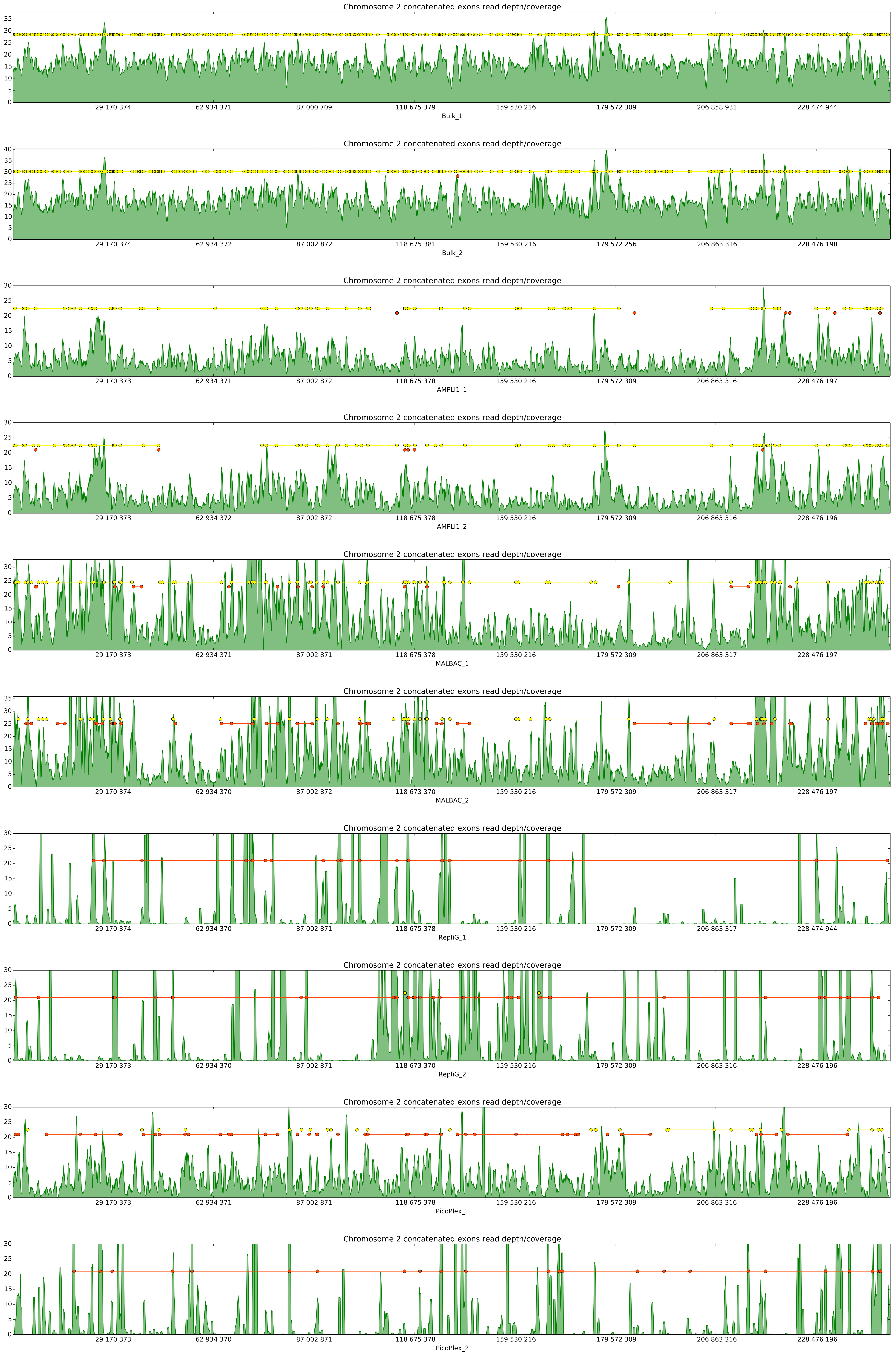

Supplementary Figure 7.

Location of the heterozygous and ADO calls in WGAs and the Bulk_1 sample on chromosome 2.
