# Supplementary figures and images for: Comparison of whole genome amplification techniques for human single cell exome sequencing
Source: PLoS One. 2017 Feb 16;12(2):e0171566. doi: 10.1371/journal.pone.0171566 (PMC5313163; doi:10.1371/journal.pone.0171566)

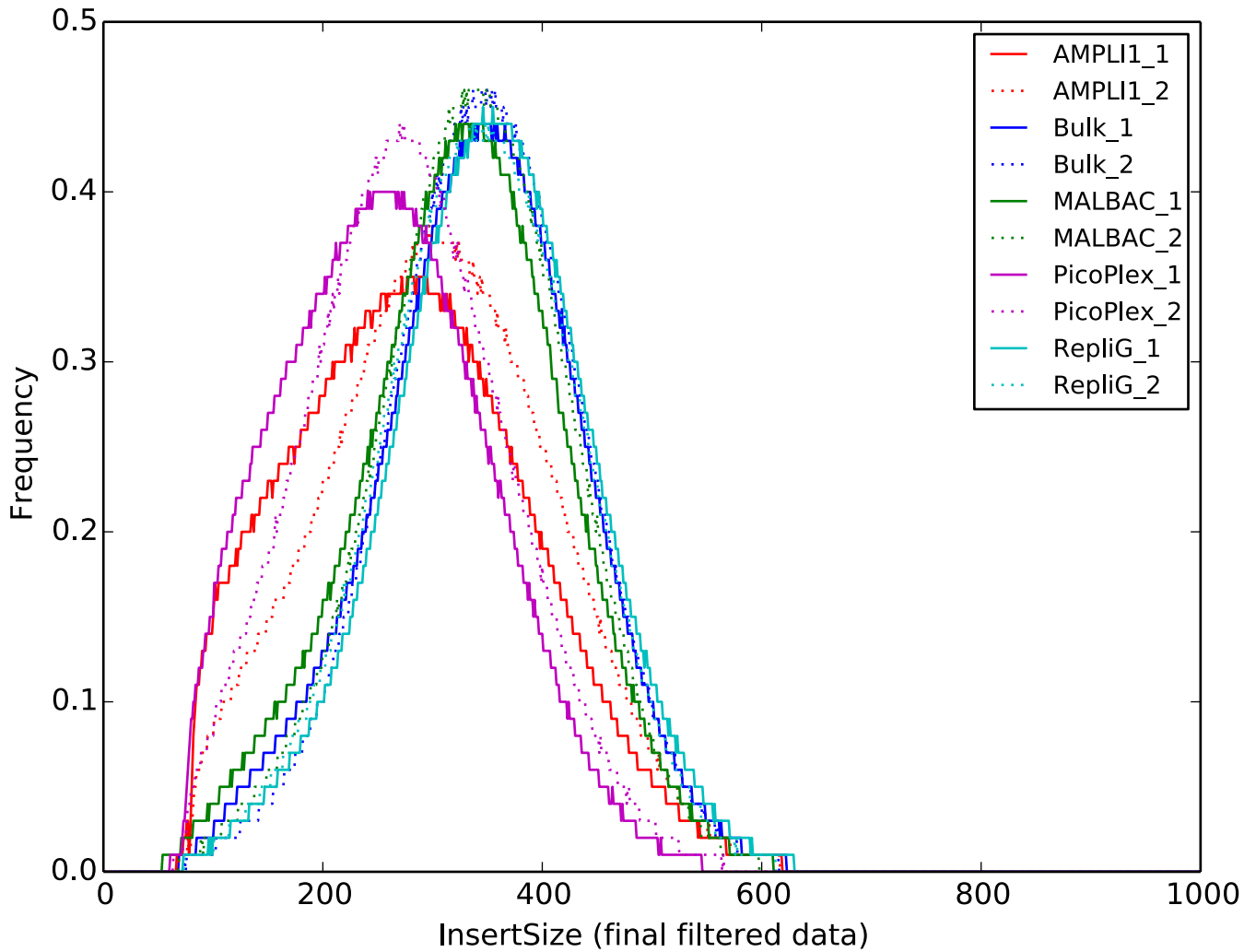

**Supplementary Figure 8.**  
Insert sizes of mapped and filtered read pairs in the 10 Million subset.

Supplement: S8 Fig — (PDF) [file pone.0171566.s008.pdf]
