# Supplementary material for: Comparison of whole genome amplification techniques for human single cell exome sequencing
Source: PLoS One. 2017 Feb 16;12(2):e0171566. doi: 10.1371/journal.pone.0171566 (PMC5313163; doi:10.1371/journal.pone.0171566)
Supplement: S1 Table — Despite identical amounts of initial material, the output data differed significantly. The target coverage was much lower for the amplified single cells (range between 7 and 68%) compared to the 90% coverage of the bulk. (PDF) [file pone.0171566.s009.pdf]

Sample ID	All data<br>Number of read pairs	Mapping Rate	Reads on target	PCR Dulpicates	Exome coverage
Bulk_1	21 365 022	99,26%	84,37%	20,59%	93,16%
Bulk_2	21 259 830	99,40%	85,92%	14,58%	92,21%
AMPLI1_1	10 605 140	92,84%	46,37%	24,59%	50,79%
AMPLI1_2	18 964 828	95,12%	52,51%	29,82%	55,21%
MALBAC_1	28 883 280	99,10%	77,30%	21,24%	68,00%
MALBAC_2	22 933 845	99,35%	85,47%	19,78%	59,37%
RepliG_1	20 594 112	99,10%	53,48%	23,81%	12,16%
RepliG_2	16 160 598	99,34%	61,88%	17,93%	21,38%
PicoPlex_1	10 617 288	94,54%	82,64%	17,48%	35,38%
PicoPlex_2	10 062 223	91,75%	83,54%	21,67%	6,95%
**Sample ID**	**10M read pairs subset**<br>Number of read pairs	Mapping Rate	Reads on target	PCR Dulpicates	Exome coverage
Bulk_1	10 000 000	99,26%	84,40%	10,94%	91,35%
Bulk_2	10 000 000	99,40%	85,88%	7,42%	90,26%
AMPLI1_1	10 000 000	92,84%	46,48%	23,74%	50,34%
AMPLI1_2	10 000 000	95,13%	54,17%	20,90%	49,75%
MALBAC_1	10 000 000	99,10%	77,92%	8,93%	59,59%
MALBAC_2	10 000 000	99,35%	85,59%	9,83%	53,53%
RepliG_1	10 000 000	99,10%	53,53%	13,80%	10,13%
RepliG_2	10 000 000	99,34%	61,89%	12,02%	19,55%
PicoPlex_1	10 000 000	94,54%	82,51%	16,69%	35,09%
PicoPlex_2	10 000 000	91,75%	83,50%	21,58%	6,96%

Supplementary Table 1.
Amount of data obtained, mapping rates, percentage of reads that map to target regions, rate of PCR duplicates and percentage of the exome covered for each sample in the full data set and the 10M read pair subset. Despite identical amounts of initial material, the output data differed significantly. The target coverage was much lower for the amplified single cells (range between 7 and 68%) compared to the 90% coverage of the bulk.
