# Supplementary material for: Comparison of whole genome amplification techniques for human single cell exome sequencing
Source: PLoS One. 2017 Feb 16;12(2):e0171566. doi: 10.1371/journal.pone.0171566 (PMC5313163; doi:10.1371/journal.pone.0171566)
Supplement: S2 Table — (PDF) [file pone.0171566.s010.pdf]

Sample Name	Untrimmed bases	PicoPlex Adapter	MALBAC Adapter	AMPLI1 Adapter	Illumina Adapter	Base Call Quality
Bulk_1	97,7%	0,0%	0,2%	0,1%	0,3%	1,7%
Bulk_2	98,1%	0,0%	0,2%	0,1%	0,3%	1,3%
AMPLI1_1	86,9%	0,0%	0,2%	10,1%	0,9%	2,0%
AMPLI1_2	87,5%	0,0%	0,2%	9,8%	0,6%	1,9%
MALBAC_1	91,6%	0,0%	6,2%	0,1%	0,3%	1,9%
MALBAC_2	95,4%	0,0%	2,4%	0,1%	0,3%	1,8%
RepliG_1	97,8%	0,0%	0,2%	0,1%	0,3%	1,6%
RepliG_2	98,2%	0,0%	0,2%	0,1%	0,3%	1,3%
PicoPlex_1	83,3%	14,0%	0,2%	0,1%	0,6%	1,8%
PicoPlex_2	84,1%	13,6%	0,2%	0,1%	0,4%	1,5%

Supplementary Table 2.
Details results of adapter and quality trimming of reads for each sample in the 10M read pair subset.
