# Supplementary material for: Comparison of whole genome amplification techniques for human single cell exome sequencing
Source: PLoS One. 2017 Feb 16;12(2):e0171566. doi: 10.1371/journal.pone.0171566 (PMC5313163; doi:10.1371/journal.pone.0171566)
Supplement: S3 Table — (PDF) [file pone.0171566.s011.pdf]

Sample Name	Fwd Rev	Fwd Fwd	Rev Fwd	Rev Rev	Different Chromosomes
Bulk_1	99,7%	0,0%	0,1%	0,0%	0,2%
Bulk_2	99,8%	0,0%	0,0%	0,0%	0,1%
AMPLI1_1	91,8%	0,5%	1,3%	0,5%	5,9%
AMPLI1_2	92,6%	0,6%	0,7%	0,6%	5,6%
MALBAC_1	99,2%	0,0%	0,1%	0,0%	0,6%
MALBAC_2	98,7%	0,0%	0,1%	0,0%	1,2%
RepliG_1	98,8%	0,4%	0,1%	0,4%	0,4%
RepliG_2	99,2%	0,3%	0,1%	0,3%	0,1%
PicoPlex_1	92,3%	0,1%	1,5%	0,1%	6,0%
PicoPlex_2	97,4%	0,1%	0,8%	0,1%	1,7%

Supplementary Table 3.
Mapping orientations of read pairs for each sample in the 10M read pair subset.
