# Supplementary material for: Comparison of whole genome amplification techniques for human single cell exome sequencing
Source: PLoS One. 2017 Feb 16;12(2):e0171566. doi: 10.1371/journal.pone.0171566 (PMC5313163; doi:10.1371/journal.pone.0171566)
Supplement: S4 Table — (PDF) [file pone.0171566.s012.pdf]

Sample	Variants called overlapping with Bulk_1		Variants with genotype call identical to the Bulk_1 genotype		
	Count	% of Bulk_1 variants	Count	% of Bulk_1 variants	Normalized for number of overlapping variants
Bulk_1	28501	100,0%	28501	100,0%	100,0%
AMPLI1_1	5239	18,4%	5049	17,7%	96,4%
MALBAC_1	8788	30,8%	7125	25,0%	81,1%
RepliG_1	1528	5,4%	792	2,8%	51,8%
PicoPlex_1	4937	17,3%	3072	10,8%	62,2%

Supplementary Table 4.
Variant and genotype calls compared to the Bulk_1 sample for the first duplicate of all samples in the 10M read pair subset.
