# Supplementary material for: Comparison of whole genome amplification techniques for human single cell exome sequencing
Source: PLoS One. 2017 Feb 16;12(2):e0171566. doi: 10.1371/journal.pone.0171566 (PMC5313163; doi:10.1371/journal.pone.0171566)
Supplement: S5 Table — (PDF) [file pone.0171566.s013.pdf]

Sample Name	Variants Called as Bulk_1		Allele Dropout	
	Count	Percentage	Count	Percentage
Bulk_1	5138	100,0%	0	0,0%
Bulk_2	4063	99,9%	3	0,1%
AMPLI1_1	730	92,5%	59	7,5%
AMPLI1_2	889	90,6%	92	9,4%
MALBAC_1	1041	81,5%	236	18,5%
MALBAC_2	722	52,5%	653	47,5%
RepliG_1	17	6,3%	251	93,7%
RepliG_2	25	4,3%	559	95,7%
PicoPlex_1	262	35,1%	485	64,9%
PicoPlex_2	4	1,6%	252	98,4%

Supplementary Table 5.
Allele dropout estimation based on variant calls compared to the Bulk_1 sample for each sample in the 10M read pair subset.
